# Characteristics and Habits of Psychiatrists and Neurologists With High Occupational Well-Being: A Mixed Methods Study

**DOI:** 10.1016/j.mayocpiqo.2024.04.005

**Published:** 2024-06-12

**Authors:** Alexis Amano, Nikitha K. Menon, Stephanie Bissonnette, Amy B. Sullivan, Natasha Frost, Zariah Mekile, Hanhan Wang, Tait D. Shanafelt, Mickey T. Trockel

**Affiliations:** a Department of Health Policy and Management, Jonathan and Karin Fielding School of Public Health, University of California, Los Angeles, CA; b WellMD & WellPhD Center, Stanford University School of Medicine, Stanford, CA; c Department of Neurology, Virginia Commonwealth University, Richmond, VA; d Mellen Center for Multiple Sclerosis, Neurological Institute, Cleveland Clinic, Cleveland, OH; e Department of Neurology, University of Wisconsin Hospitals and Clinics, Madison, WI; f PGSP-Stanford Psy.D. Consortium, Palo Alto University, Stanford University, Palo Alto, CA

## Abstract

**Objective:**

To identify the characteristics that distinguish occupationally well outliers (OWO), a subset of academic psychiatrists and neurologists with consistently high professional fulfillment and low burnout, from their counterparts with lower levels of occupational well-being.

**Participants and Methods:**

Participants included faculty physicians practicing psychiatry and neurology in academic medical centers affiliated with the Professional Well-being Academic Consortium. In this prospective, longitudinal study, a mixed qualitative and quantitative approach was used. Quantitative measures were administered to physicians in a longitudinal occupational well-being survey sponsored by the academic organizations where they work. Four organizations participated in the qualitative study. Psychiatrists and neurologists at these organizations who competed survey measures at 2 consecutive time points between 2019 and 2021 were invited to participate in an interview.

**Results:**

Of 410 (213 psychiatrists and 197 neurologists) who completed professional fulfillment and burnout measures at 2 time points, 84 (20.5%) met OWO criteria. Occupationally well outliers psychiatrists and neurologists had more favorable scores on hypothesized determinants of well-being (values alignment, perceived gratitude, supportive leadership, peer support, and control of schedule). Ultimately, 31 psychiatrists (25% of 124 invited) and 33 neurologists (18.5% of 178 invited) agreed to participate in an interview. Qualitatively, OWO physicians differed from all others in 3 thematic domains: development of life grounded in priorities, ability to shape day-to-day work context, and professional relationships that provide joy and support.

**Conclusion:**

A multilevel approach is necessary to promote optimal occupational well-being, targeting individual-level factors, organizational-level factors, and broader system-level factors.

Physician well-being is a recognized health care quality indicator.^1^, ^2^, ^3^ Its counterpart, physician burnout has received attention as a salient concern for over a decade^4^, ^5^, ^6^ and is associated with increased medical errors, unsolicited patient complaints, turnover, and costs of care.^5^, ^6^, ^7^, ^8^, ^9^, ^10^, ^11^, ^12^, ^13^, ^14^, ^15^ A growing number of organizations are focusing on physician professional fulfillment as an aspirational goal warranting intervention.^16^, ^17^, ^18^, ^19^ Recent studies indicate that low professional fulfillment and high burnout are both independently associated with physicians’ intentions to leave the organizations where they practice medicine.^20^^,^^21^ In this context, psychiatrists and neurologists are similar specialties, experiencing very similar rates of burnout and intent to leave in recent studies.^20^^,^^22^ Furthermore academic faculty physicians in these specialties may be particularly vulnerable to burnout owing to the need to manage teaching, research, and academic responsibilities in addition to their clinical work.^23^ Throughout the coronavirus disease (COVID-19) pandemic, these challenges intensified.^24^, ^25^, ^26^

Understanding the factors associated with high levels of occupational well-being specific to these specialties is crucial for developing targeted strategies to promote overall well-being.^27^, ^28^, ^29^, ^30^, ^31^ In this mixed methods analysis, we aimed to identify distinctive features that differentiate psychiatrists and neurologists who consistently exhibit high professional fulfillment and low burnout from their peers.

## Participants and Methods

### Study Sample

The Professional Well-being Academic Consortium (PWAC) is a self-funded group of academically affiliated health care organizations engaged in regular longitudinal assessment of clinician well-being.^32^ Nine organizations administered the PWAC survey twice between 2019 and 2021. Previously collected, deidentified data from both survey administrations at each site were used in this study. Four sites participated in the qualitative assessment.

### Quantitative Measures

Professional Well-being Academic Consortium requires participating organizations to include the Professional Fulfillment Index in their wellness surveys, which is a measure designed to assess burnout and professional fulfillment in clinicians.^33^ The chief wellness officers and other clinician wellness experts representing each participating PWAC discus and vote on which measures of hypothesized determinants of clinician well-being to prioritize. Measures are selected from domains of personal resilience (individual clinician factors), culture of wellness, and efficiency of clinical practice.^34^ Many of these survey measures found association with clinician burnout and professional fulfillment in previously published literature, including the following: personal-organization values alignment, self-valuation, sleep-related impairment, perceived gratitude, and impact of work on personal relationships, and supportive leadership.^10^^,^^15^^,^^35^, ^36^, ^37^, ^38^ Measures of peer support, perceived control of work schedule, electronic health record hassles, and electronic health record helpfulness were used as well. The associations of these later 4 measures with burnout and professional fulfillment have not been published to our knowledge. Quantitative survey measures and the scales they are derived from can be found in Supplemental Material 1 (available online at http://www.mcpiqojournal.org).

### Quantitative Analysis Methods

Consistent high occupational well-being was defined by high professional fulfillment and low burnout at both survey points. To evaluate the characteristics of this occupationally well outlier (OWO) group, we calculated the pooled standard deviation (SD) unit differences between this group and all others, referred to as predominant wellness level (PWL) group, in scores on measures of hypothesized determinants of well-being. We calculated group differences before and after adjusting for specialty, gender, and age category because these variables have been associated with physician occupational well-being.^22^ Other demographic variables were not available owing to data deidentification. We focused on large, standardized differences (Δ ≥ 0.8 SD units) between groups and determined statistical significance of group differences using independent samples *t* tests. Statistical significance was set at 2-tailed *P*<.05.

### Qualitative Assessment

Eligible participants for the qualitative assessment included faculty psychiatrists and neurologists who completed 2 PWAC surveys and agreed to share their survey data. Those eligible were offered a $200 incentive for participation. Interview participants were categorized into the comparator groups described earlier: OWO and PWL groups.

Between November 2020 and July 2021, 2 trained interviewers (including N.K.M.) conducted 60-minute, semistructured virtual interviews with 64 psychiatrists and neurologists (additional details in Supplemental Material 2, available online at http://www.mcpiqojournal.org). Interviews explored factors that participants felt prevented or ameliorated burnout and drove high professional well-being. Each interview was followed by a brief demographic survey. All interviews were recorded and transcribed verbatim (GoTranscript; Athreon). Transcripts were paired with their respective demographic and longitudinal occupational well-being data to enable qualitative comparison. Data collection continued until thematic saturation was reached.

We conducted a multistep thematic analysis of interviews. A single author (N.K.M.) and a qualitative expert created an initial codebook based on emergent themes from early transcripts using a constant comparative method within the software (Dedoose Version 8.3.47) to categorize the remaining data. A subset of transcripts was reviewed to reach consensus on a coding structure. Subsequently, all remaining transcripts were coded independently (N.K.M., Z.M., A.A.), with a subset coded in sequence to ensure consistency. Following this stage of data disassembly, data reassembly involved the analysis of each code to identify patterns to support context analysis. Analysis focused on unique characteristics of OWO physicians compared with those of PWL.

Organizational affiliations were masked, gender pronouns were randomly assigned, and details were excluded where they may compromise interviewee identity. Informed consent was obtained for all interviewees. All aspects of the study were reviewed by the Stanford Institutional Review Board.

## Results

At time 2, 2761 psychiatrists and 539 neurologists responded to the PWAC survey in which 19,029 practicing physicians participated (of 37,511 invited; response rate = 50.7%). Of these, 219 psychiatrists (28.8%) and 200 neurologists (37.1%) also provided data at time 1 (Table 1); 410 (213 psychiatrists and 197 neurologists) provided professional fulfillment and burnout data at both time points. Of these, 84 (20.5%) had high professional fulfillment and low burnout at both time points, qualifying as OWO. Compared with PWL, OWO physicians had more favorable scores on all measures of hypothesized determinants of well-being at both time points (Table 2).^39^

**Table 1:** Demographic Characteristics of Psychiatrists and Neurologists in This Study

N	Level	Overall, n (%)
		419
Primary medical specialty	Neurology	200 (47.7)
	Psychiatry	219 (52.3)
Gender	Female	211 (50.4)
	Male	202 (48.2)
	Missing	6 (1.4)
Age (y)	30-39	99 (23.6)
	40-49	149 (35.6)
	50-59	92 (22)
	60 or older	70 (16.7)
	Missing	9 (2.1)
Ethnicity	Hispanic/Latino	15 (3.6)
	Not Hispanic/Latino	355 (84.7)
	Missing/question not included in survey	49 (11.7)
Race	African American or Black	13 (3.1)
	American Indian or Alaska Native	0 (0)
	Asian	54 (12.9)
	Native Hawaiian or Pacific Islander	0 (0)
	White (European, Middle Eastern, Other)	289 (69)
	Prefer to self-describe	20 (4.8)
	Multiple Races Selected	5 (1.2)
	Missing/Question not included in survey	38 (9.1)
Sexual orientation	Prefer not to say	14 (3.3)
	Bisexual	7 (1.7)
	Gay or Lesbian	12 (2.9)
	Queer	3 (0.7)
	Straight	194 (46.3)
	Prefer to self-describe	1 (0.2)
	Missing/question not included in survey	188 (44.9)

**Table 2:** Psychiatrists and Neurologists With High Occupational Well-Being Compared With Predominant Well-Being Level Physicians^a^

Aligned theme	Hypothesized determinant of occupational well-being	Scale that measure is derived from	Time 1				Time 2			
			Unadjusted difference		Adjusted^b^ difference		Unadjusted difference		Adjusted^b^ difference	
			ES^c^	*P*^d^	ES^c^	*P*^d^	ES^c^	*P*^d^	ES^c^	*P*^d^
Building a life grounded in personal priorities	Personal-organization Values alignment	Stanford Values Alignment scale^35^	**0.94**	<.001	**0.87**	<.001	**1.13**	<.001	**1.10**	<.001
	Self-valuation	Clinician Self-valuation Scale^36^	0.76	<.001	0.68	<.001	**0.90**	<.001	**0.83**	<.001
	Sleep-related impairment	PROMIS Sleep-Related Impairment^39^	−0.57	<.001	−0.53	<.001	−**0.88**	<.001	−**0.82**	<.001
Ability to shape day-to-day work context	Electronic health record helpfulness	Stanford health care worker measurement system, unpublished	0.33	.01	0.39	.003	0.40	.001	0.49	<.001
	Electronic health record hassles	Stanford health care worker measurement system, unpublished	−0.56	<.001	−0.57	<.001	−0.43	.002	−0.42	.003
	Control of schedule	Stanford health care worker measurement system, unpublished	**0.82**	<.001	0.71	<.001	0.77	<.001	0.70	<.001
Professional relationships that provide joy and support	Supportive leadership	Mayo Clinic Participatory Management Leadership Index^38^	0.52	<.001	0.56	<.001	0.63	<.001	0.68	<.001
	Perceived gratitude	Gratitude at Work Inventory^37^	**0.81**	<.001	**0.81**	<.001	0.64	.001	0.73	<.001
	Peer support	Stanford health care worker measurement system, unpublished	**0.82**	<.001	**0.83**	<.001	**1.05**	<.001	**1.05**	<.001
	Impact of work on personal relationships	Stanford Impact of Work on Personal Relationships Scale^15^	−0.67	<.001	−0.60	<.001	−**0.90**	<.001	−**0.89**	<.001

PROMIS, Patient-Reported Outcomes Measurement Information System.

a Psychiatrists and neurologists with high occupational well-being: n=84; others: n=326.

b Adjusted for gender, age category, and psychiatry vs neurology specialty.

c Effect sizes (ES) estimated by dividing the mean difference by the pooled standard deviation (SD). ES > 0.8 SD are bolded.

d *P* values are from 2 independent sample *t* test.

Of the 410, 302 (124 psychiatrists, 178 neurologists) were associated with sites participating in the qualitative assessment and were invited to participate in an interview. Ultimately, 31 (25%) psychiatrists and 33 (18.5%) neurologists agreed to participate (Table 3). Fourteen of these respondents qualified as OWO (11 neurologists, 3 psychiatrists). Qualitatively, OWO physicians differed from PWL in 3 domains: development of a life grounded in personal priorities, ability to shape day-to-day work context, and professional relationships that provide joy and support. All physicians interviewed expressed a desire to develop these areas, but OWO physicians actualized these aspirations while PWL physicians described challenges doing so (Table 4).

**Table 3:** Demographic Characteristics of Interview Respondents

Qualitative study sample			
Qualitative interview (% of invited sample)	33 (19% of 178)	31 (25% of 124)	64 (21.2% of 302)
OWO (% of interviewed participants)	11 (33% of 33)	3 (10% of 31)	14 (4.6% of 302)
Interview sample: self-reported demographic and practice characteristics			
Qualitative Interview Participants	Neurologists	Psychiatrists	Total
N (%)	33 (51.6)	31 (48.4)	64 (100)
Gender			
Female	20 (58.8)	14 (41.2)	34 (53.1)
Male	13 (43.3)	17 (56.7)	30 (46.9)
Age (y)			
29 or younger			
30-39	6 (46.2)	7 (53.8)	13 (20.3)
40-49	15 (55.6)	12 (44.4)	27 (42.2)
50-59	3 (42.9)	4 (57.1)	7 (10.9)
60 or older	5 (41.7)	7 (58.3)	12 (18.8)
Missing	4 (80)	1 (20)	5 (7.8)
Race			
African American or Black	0	0	-
American Indian or Alaska Native	0	0	-
Asian	12 (54.5)	10 (45.5)	22 (34.4)
Native Hawaiian or Pacific Islander	0	0	-
White (European, Middle Eastern, and other)	18 (50)	18 (50)	36 (56.3)
Missing	3 (75)	1 (25)	4 (6.3)
Multiple races selected	0	2 (100)	2 (3.1)
Total years in practice, including residency			
More than 20 y	7 (35)	13 (65)	20 (31.3)
16-20 y	5 (45.5)	6 (54.5)	11 (17.2)
11-15 y	8 (66.7)	4 (33.3)	12 (18.8)
5-10 y	8 (61.5)	5 (38.5)	13 (20.3)
Less than 5 y	1 (33.3)	2 (66.7)	3 (4.7)
Marital status			
Divorced	1 (50)	1 (50)	2 (3.1)
Married	27 (51.9)	25 (48.1)	52 (81.3)
Separated	1 (50)	1 (50)	2 (3.1)
Single	3 (42.9)	4 (57.1)	7 (10.9)
Missing	1 (100)		1 (1.6)

OWO, occupationally well outlier.

**Table 4:** Additional Quotes Illustrating Differences in Occupational Well Outliers and Predominant Wellness Level Physicians

Theme	Subtheme	OWO physicians	PWL physicians
Building a life grounded in personal priorities	Identifying life priorities	“…you just have to accept that your trajectory is going to be a little slower academically and also that you are not going to be quite the perfect parents you'd want to be, but you have to compromise a little on each end… that was something that I think really helped me make peace of the fact that maybe I wasn't as ambitious or successful as the person in the office next to me, but that I was doing these other things with family and children that were important. I would say that probably having children has been the most important thing. I would rank it just as or probably more important than my career.” “I think just accepting what is, that you can't be everywhere at the same time and just trying to be present in the place that you've decided that you're going to be in that moment. If you've decided that you're going to be at work and you're going to have to miss the recital, you might as well be present at work and not at work and feeling bad the whole time and not doing a good job at work or vice versa. If you decide to leave work early with none of your notes and none of your MyCharts done, go enjoy the soccer game and just know that when you get home, you're going to have to buckle down and get all that work done that you didn't do at the end of the day”	[No quotes for this subtheme]
	Harmonizing personal and professional life	“I know that’s a hot topic in a way and protecting your personal time. To me, they really blend together, and I like it that way. I'll be not on call at all for anything, but a resident will have some crisis and call me, and they all have my cell phone and I encourage them to call, and it's fine.… My life and my work are all mish-mashed together…To me maintaining that balance isn't a challenge. There is none, and I don't mean that I don’t care in the sense that I’m all work all the time, I just mean that it is inherently balanced…It’s probably not for everybody, but I’m actually perfectly happy with it” “I would try to have this rule that when I was at work I was completely available to see the patients. I would work pretty much the whole day often with very short lunch breaks. I just wanted to get all the work done, but at the end of the day that was it…and I could go to the gym or do other things. So I think it was helpful having that boundary between work and home.”	“Patient care is hard to balance, because so much of it, you get messages at 4 PM. When you're doing clinical care, most of your work is from 4 to 7, because that's when people contact you. If you wait till the next day, it just starts to pile up. The urgent need to respond to people for clinical care makes it really, really difficult.” “…this EMR follows you everywhere and the expectations are rising and it’s very hard to establish boundaries” “…I think it’s very easy when your office is your home, to stay up late and do notes because you can, instead of putting it away…I just think we just have to try a little bit harder to separate the two, because otherwise, it could get very easy to be overcome by it.” “…people choose to be at [FACILITY] for the privilege and the joy and the pleasure of engaging in academic work, but when the work week gets extended to the point where you’re working almost an unsustainable number of hours during the day and through the weekend, and you have to cut back on those things, and then it does force a reckoning of well, is academia really sustainable then.”
	Accepting human limitations and a growth mindset	“Physicians tend to be very perfectionistic, they tend to feel guilty very easily and they have an exaggerated sense of responsibility. This triad is not good. It's not good at all…. You have to be able to say to yourself, ‘I've done a good job, it's good enough.’ … Of course, you don't want any of your patients to die because of negligence, but we all make mistakes. Mistakes are not pervasive, mistakes are not permanent. How do you learn from that and how do you move on, I think that's a big part of being a physician.” “I connect with my patient and I disconnect. I don't attach because once you have attachment, then that detachment is painful. I never try to attach with my patient. I always have a connection because that connection is invisible, but attachment can become painful once you detach yourself. I feel that I need to be connected, not attached…”	“I think that I’m always thinking about my patients to some degree” “…you’re never off and even when you’re off, maybe you’re ruminating about mistakes you might’ve made or things you could have done better, or did you forget something or trying to figure out an enigmatic pace or something like this. I think just the consumption of the profession, in all aspects, is really something that I think you just have no clue about when you start.” “I’ve had numerous patients commit suicide, so now when a patient commits suicide, there’s always that small voice in the back of my head that questions what I might have not done or what I might have not done well enough to potentially prevent his suicide. It's something that unique to psychiatry.”
Ability to shape day-to-day work context	Lowering the intensity of work	“…you have to be your best cheerleader, and if you don’t bring things up, nothing’s going to change. With our schedule, we were burning ourselves to death seeing all of these patients, making the assumption that everybody knew how bad we were struggling. As soon as we brought it up, we had the CEO of the hospital calling us saying, ‘Why didn’t you let us know that you were struggling?’… They made some changes in our daily schedule to… give us extra time to catch up.” “I’ve been able to build the programs that I’ve run... in ways that ensured, I do not know, sustainability,…I think part of the issue there is that a lot of that stuff that adds to the workload compression and stress and burnout isn't reimbursable. They just throw it all together, and so it looks like you're doing X amount of work because this is how many patients you saw in the hospital, but you're really doing X plus Y plus Z amount of work…From the bean counters perspective, you did X, but from your perspective you did X, Y, and Z and didn't get credit for it, and so you get burnout. What I did was I made sure that we separate out X, Y, and Z, and everybody’s doing these things in a way that it's not necessarily easy, but it's sustainable and the hospital just pays for it, and they're not thrilled about it. I just keep making the argument that you wouldn’t expect a supplier to give you free stuff…”	“I think just earlier recognition of what a physician’s value is, how they practice and what’s going to make their practice most successful in the way that they would like to practice, rather than squeezing every physician into a preset box. What do you envision your clinic looking like; how do you like this; how would you like this to run. Because everyone’s a little different…that autonomy piece that I brought up is probably the most often ignored by institutions” “There’s certain things I’ve asked for for years now that still haven’t happened…I’d say, a third of the time squawking helps; two-thirds of the time you’re just squawking to somebody else who says, ‘I recognize the problem. I'm sorry.’”
Professional relationships that provide joy and support	Supportive culture at work	“The junior faculty are very tight with each other. They're like, I call it like pig-pen. Pig-pen has like the clump of dirt like hanging around and they all just hang out together, like kids around a soccer ball. I love it.” “It’s not like you can just change your culture on a dime…your leadership has to buy into it, not just the chair, but the administrative director, the vice-chairs, the division chiefs, they all got to drink the Kool Aid and be consistent with this…We put a lot of emphasis in our division chiefs and our leaders, this is how we lead and this is what we truly believe in…life is too short not to be touchy-feely and to really care about the people you work with.”	“For my work life to be a little bit happier, I think there is a little bit of a pecking order at my institution. It's established who's junior and who's senior. I think trying to continue to get publications and get my own independent funding would help me feel more established and have more of a footing in the hierarchy of the institution. I feel like the people that get taken rather more seriously are the ones that are leading clinical trials or have multimillion dollar funding. That creates somewhat of a dynamic between different people in the institution. Overall, people are very collegial, but I think it’s still a hidden but known presence there.” “I think people are still very much concerned about their image and what their colleagues think of them and, ‘Is this going to affect my climb up the ladder?’ and all that stuff. There may be some validity to that. I don’t know how the department would look at someone who comes forward to say that they’re struggling.” “You are not accepted if you detach…Everybody thinks it’s so amazing that I take time off, or it’s amazing that I don’t give my cell phone number. They think I am this weird freak of a person. They don't set an away message on their emails when they leave. They don't have somebody cover their inbox. It's, to me, a total nightmare…they’re pure workaholics, and it’s set by the chief.” “…being in psychiatry, people are usually mindful of these things, so the way that messages are given more explicitly is ‘Yes, take time off whenever you need it; reach out to us whenever you need it.’ There wasn’t always action…No one ever said, don’t take time off, but there’s always been this expectation that if you don’t do it, who else is going to do it? There are some people in leadership who have made comments that, ‘If people don't want to work hard, we don't want them at [LOCATION].’”
	Interpersonal relationships at work that provide joy and support	“I do think that the human interaction, whether it’s their family or other friends, that’s essential in being able to keep up with your work, and a good environment at work…it makes a difference in your everyday life…” “…I really enjoy being in the operating room, I like talking to the surgeons, the anesthesiologists, scrub techs, my intraoperative monitoring techs. That whole in-person interaction is highly valuable…there’s kind of an intangible niceness to having the interactions in-person.” “…it’s just almost like therapy in a way. It is just someone to talk to, work through the problem, sometimes bounce ideas off people. Misery loves company, so somehow there is some comfort in knowing that you're not the only one going through it. I think all those things. You can relate to someone…it may not be exactly the same but just there’s enough commonalities usually that there’s some reassurance it’s normal.” “…our ‘team’ from techs to RN’s to APP’s and other faculty. I like that we really act and feel like a team. That helps us take on more and we can support each other. We laugh a lot together and that really helps us through the more depressing and stressful times.” “Everybody is supportive in my group. When I needed a day off in June, because I was burned out, I tell them, ‘Can somebody cover my call for the day?’ They say, ‘Yes, sure, no problem.’”	“…for a while I felt lack of support, that I didn’t have a team that was big enough to cover the amount of stuff that I was doing…It used to be that Wednesday and Friday clinic would bleed so much into the other days that I was like, ‘How is this possible that I can’t get anything done?’” “It’s tricky because I think it’s harder to maintain that social collegial atmosphere. Conversations are much more transactional. You're getting on Zoom to solve a problem and not just to check in. I think what has really been missed is all that before and after the meeting small talk, 'cause you can't really small talk in a group meeting cause everybody’s listening to everybody. Just like, ‘Hey, how is it going? What is happening? Show me a picture of your kids.’ All that kind of stuff that keeps people interpersonally connected is really necessary and really hard to recreate.”

APP, advanced practice provider; CEO, chief executive officer; EMR, electronic medical record; OWO, occupationally well outliers; PWL, predominant wellness level; RN, registered nurse.

### Building a Life Grounded in Personal Priorities

All physicians pursued lives informed by their priorities. OWO physicians did so by identifying their priorities then making intentional decisions about work life integration. Some OWO physicians reported that accepting human limitations and adopting a growth mindset supported boundary setting. Predominant wellness level physicians experienced difficulties placing boundaries on their time and emotions, challenging their ability to build a life based on priorities.

Quantitative measures conceptually related to this theme included personal-organizational values alignment, self-valuation, and sleep-related impairment (Table 2). Differences in personal-organizational values alignment between OWO physicians and PWL were large (Δ ≥ 0.8 SD units; *P*<.001) at both times. Additionally, self-valuation and sleep-related impairment data reported large favorable differences (Δ ≥ 0.8 SD units; *P*<.001) in OWO physicians compared with those in PWL at time 2 and moderate favorable differences (Δ ≥ 0.5 SD units; *P*<.001) at time 1.

#### OWO: Identifying Priorities

OWO physicians emphasized the importance of identifying their priorities before making career decisions. One physician expressed that doing so enabled her to build a career that honors her priorities: “I think having a sense of knowing what I wanted in my life and really going after that and trying not to deviate too far from that because there were lots of pulls of different ways my career had gone…” (OWO). Importantly, OWO physicians highlighted that these priorities can and do change.

On identifying their priorities, OWO physicians described making intentional choices about their desired careers. One physician noted that because “having children has been the most important thing,” she has accepted that her “trajectory is going to be a little slower academically…but that [she] was doing these other things with family and children that were important” (OWO). Few physicians in the PWL group discussed defining their life priorities.

#### OWO: Harmonizing Personal and Professional Life

OWO physicians described integrating their professional and personal lives in a way that best serves their goals and lifestyle. For 1 physician, this entailed purposefully blending his professional and personal life: “I know that's a hot topic in a way and protecting your personal time. To me, they really blend together, and I like it that way” (OWO). Most, however, described setting clear boundaries between their professional and personal life: “…once your work is done, you should go home…I don't think about anyone [patients or colleagues] when I'm home” (OWO).

Harmonizing personal and professional life also involved setting emotional boundaries. OWO physicians described building empathetic but bounded relationships with patients and their families: “I connect with my patient, and I disconnect. I don't attach because once you have attachment, then that detachment is painful…” (OWO).

#### PWL: Barriers to Harmonizing Personal and Professional Life

Predominant wellness level physicians described a desire to harmonize their personal and professional lives but reported that responsibilities that extend into personal time and difficulty releasing emotional stressors from work challenged their ability to do so.

Clinical and research tasks that extended into personal time included patient messages, documentation, and professional opportunities at academic medical institutions. Additionally, physicians reported that the growing omnipresence of the electronic medical record and the shift to work-from-home have both increased the ease with which work can creep into personal time.

Physicians also faced challenges setting emotional boundaries. One physician expressed, “I think that I'm always thinking about my patients to some degree” (PWL). Some felt that the emotional burdens they experienced were unique to psychiatrists and neurologists, including caring for patients who commit suicide: “…when a patient commits suicide, there's always that small voice in the back of my head that questions what I might have not done…” (PWL).

#### OWO: Accepting Human Limitations and Adopting a Growth Mindset

OWO physicians described the importance of adopting a growth mindset: “…we all make mistakes…. How do you learn from that and how do you move on, I think that's a big part of being a physician” (OWO). OWO physicians also noted that setting emotional boundaries requires accepting what is outside of a physician’s control: “Sometimes there is sadness because you see the outcome doesn't get better. Then I also remind myself that I shouldn't be focusing on an outcome… I'm not the ultimate result-giver…” (OWO).

#### PWL: Barriers to Accepting Human Limitations and Adopting a Growth Mindset

Predominant wellness level physicians described challenges accepting human limitations and adopting a growth mindset. Some reported “ruminating about mistakes you might've made or things you could have done better” (PWL). Several physicians expressed a desire to “let go of” past mistakes but found it difficult to do so (PWL).

### Ability to Shape Day-to-Day Work Context

OWO physicians described greater perceived ability to make their work systems more ideal for themselves and their colleagues. Predominant wellness level physicians did not often describe having as much workplace autonomy or agency.

Quantitative measures related to this theme included control of schedule as well as electronic health record hassles and helpfulness. Control of schedule was more favorable by a large effect size difference at time 1 before but not after adjusting for demographic variables. Neither electronic health record measure reported large effect size differences in OWO physicians vs PWL physicians.

#### OWO: Lowering the Intensity of Work

OWO physicians were able to lower the intensity of work. Some reported raising awareness about and advocating for physician needs. OWO physicians in leadership roles described efforts to improve the workplace for their colleagues. One physician designed a department with the intent of supporting physician well-being. Specifically, he ensured that the hospital compensates physicians for all the work they do, including nonreimbursable work.

#### PWL: Barriers to Lowering the Intensity of Work

Predominant wellness level physicians described greater difficulty making changes to their work context. Many desired a greater understanding of physician needs at work. One physician highlighted that the physician “…autonomy piece…is probably the most often ignored” in the workplace (PWL). Predominant wellness level physicians noted that even when physician needs are raised, they are not always met.

### Professional Relationships That Provide Joy and Support

The final distinguishing area between OWO and PWL physicians was the professional relationships they described. Interpersonal relationships were important to all physicians; however, physicians in the PWL group faced barriers to cultivating them. Importantly, culture at work was described as an antecedent to interpersonal relationships. OWO physicians described more positive, supportive professional cultures than PWL physicians.

Quantitative measures aligned with this theme included supportive leadership, perceived gratitude, peer support, and impact of work on personal relationships. Differences between OWO physicians and PWL that were large (Δ ≥ 0.8 SD units; *P*<.001) were perceived gratitude and peer support at time 1 and peer support and impact of work on personal relationships at time 2.

#### OWO: Supportive Culture at Work

Workplace culture was identified by all physicians as a precursor to interpersonal relationships. OWO physicians described a supportive workplace culture. Some expressed that building a positive culture requires consistency and follow through from leaders: “It's not like you can just change your culture on a dime…your leadership has to buy into it…and be consistent….” (OWO).

#### PWL: Barriers to a Supportive Culture at Work

Some in the PWL group also reported a positive culture at work; however, most described implicit factors that challenged relationship building, the discussion of burnout, and boundary setting.

Some physicians described a “pecking order” at their institutions that challenged relationship building (PWL). One physician reported that at his institution, “the people that get taken more seriously are the ones leading clinical trials or have multimillion dollar funding,” creating a “hidden but known presence” (PWL).

Additionally, many physicians reported outward support for burnout at their institutions but lingering concerns about the repercussions of discussing burnout, including negative perceptions from colleagues and challenges advancing in their career. One physician noted, “I don't know how the department would look at someone who…[says] that they're struggling” (PWL).

Predominant wellness level physicians felt that the culture of their department did not support boundary setting. One physician felt like the “black sheep” in her department for setting boundaries: “Everybody thinks it is so amazing that I take time off… They think I'm this weird freak…” (PWL).

#### OWO: Interpersonal Relationships at Work that Provide Joy and Support

OWO physicians described professional relationships that provide joy and support. One physician expressed, “… the human interaction… that's essential in being able to keep up with your work…” (OWO).

Some found that colleague relationships provided a venue to discuss and process shared experiences, “…it’s just almost like therapy in a way…” (OWO). Relationships with colleagues were also found to provide emotional and professional support during difficult periods. One physician described, “I like that we really act and feel like a team. That helps us take on more and we can support each other. We laugh a lot together and that helps us through the more depressing and stressful times” (OWO). Another physician was easily able to find shift coverage during a period of burnout.

#### PWL: Barriers to Interpersonal Relationships at Work that Provide Joy and Support

Physicians in the PWL group valued social interactions at work but faced challenges building or receiving support from these relationships. Barriers cited included a lack of resources to support team members and the dispersion of colleagues due to COVID-19 work-from-home practices.

Some felt that even when they had positive interpersonal relationships with teammates, their teammates were unable to support them during challenging periods. One physician noted that her biggest challenge was “that I didn't have a team that was big enough to cover the amount of stuff that I was doing” (PWL).

Many in this group felt that interpersonal relationships had weakened owing to the shift to remote work during the COVID-19 pandemic. Some described a loss of social interactions that challenged relationship building and maintenance: “…what has really been missed is all that before and after the meeting small talk…that kind of stuff that keeps people interpersonally connected is really necessary and really hard to recreate” (PWL).

## Discussion

We observed several factors that characterize OWO psychiatrists and neurologists. In terms of the quantitative factors, 2 of the 5 assessed culture of wellness factors (personal-organization values alignment and peer support) were more favorable by a large margin at both time points. All 3 personal resilience factors assessed (self-valuation, sleep-related impairment, and impact of work on personal relationships) also differentiated OWO physicians by a large margin.

Our qualitative findings largely mirrored group differences in quantitative measures and illustrate the meaning of these measures in the lived experience of psychiatrists and neurologists. However, our findings also indicate opportunities for additional quantitative assessment. Congruent measures included the self-valuation measure, which assesses a physician’s degree of self-compassion and self-care, strongly paralleled the qualitative subtheme—accepting human limitations and adopting a growth mindset. Both the quantitative and qualitative findings highlight the importance of self-compassion in physician well-being. In interviews, OWO physicians articulated that they do not ultimately control their patients’ outcomes and can make mistakes. As such, self-compassion is an integral precursor to OWO. Qualitative data also illustrated the experience of peer support and the importance of interpersonal relationships at work.

A qualitative theme that did not have a congruent quantitative measure was the ability to lower the intensity of work. This theme extends beyond physicians’ ability to control their schedule and may provide a deeper understanding of their agency and autonomy at work. Agency and voice in determining pace and process of work may be essential ingredients to OWO.^40^

Overall, our findings indicate that a multilevel approach, including individual-level, organizational-level, and system-level factors, is necessary to address occupational well-being. Physicians highlighted the impact of both the individual factors that they can control as well as the workplace factors they cannot control on their well-being. Individual factors mentioned included intentionally integrating personal and professional life as well as accepting human limitations and adopting a growth mindset. To support individual well-being, organizations can implement interventions such as professional coaching to support physicians in developing self-valuation (also known as self-compassion).^41^ Organizational-level factors included physicians’ ability to shape their day-to-day work context and develop professional relationships that provide joy and support. Importantly, physicians’ ability to thrive was not solely a product of their own abilities; institutional factors also played a major role. As such, institutions have the potential to address well-being by designing workflows in a way that promotes sustainability and flexibility. Participatory management models, such as Listen-Act-Develop, which engages physicians in system improvement, may be a way to develop systems that support physician agency and autonomy.^35^^,^^38^^,^^40^ Systems can also promote the development of professional relationships through interventions such as commensality groups.^42^ In addition, our findings indicate that the leaders within these systems matter. Leadership that considers frontline needs and workplace culture can support physician well-being and result in better health care systems.^43^^,^^44^

## Limitations

Study generalizability is likely limited by the timing of data collection during the COVID-19 pandemic. Greater clinical demands during the pandemic may have precluded some from accepting our study invitation. Additionally, completion of 2 surveys was a prerequisite to recruitment for qualitative interviews, limiting our sample. Furthermore, respondents who identified their race were either White or Asian. Thus, our findings may not be generalizable to groups underrepresented in medicine. Finally, associations observed in quantitative analyses cannot determine causal relationships.

## Conclusion

This longitudinal, mixed methods assessment of psychiatrist and neurologist occupational well-being identified differentiators between OWO and their colleagues. These factors may be helpful in prioritizing and developing strategies to improve occupational well-being among physicians and include the ability to build a life grounded in personal priorities, the ability to shape day-to-day work context, and the presence of professional relationships that provide joy and support.

## Potential Competing Interests

Dr Trockel receives payments for consulting services provided to Marvin Behavioral Health and also honoraria payments for some of the keynote presentations and grand rounds at various healthcare organizations. The other authors declare no competing interests.
