# Supplementary material for: Characteristics and Habits of Psychiatrists and Neurologists With High Occupational Well-Being: A Mixed Methods Study
Source: Mayo Clin Proc Innov Qual Outcomes. 2024 Jun 12;8(4):329–42. doi: 10.1016/j.mayocpiqo.2024.04.005 (PMC11223072; doi:10.1016/j.mayocpiqo.2024.04.005)
Supplement: Supplemental Material [file mmc1.pdf]

I. Quantitative Data Collection Instrument

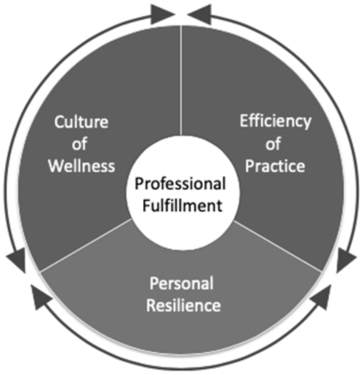

The Stanford Model of Professional Fulfillment ™
The PWAC survey organizes hypothesized determinants of occupational well-being into three categories based on the Reciprocal Model of Physician well-being (aka the Stanford Model of Professional Fulfillment).[1] These categories are "Culture of Wellness" (for the current study: peer support, control of schedule, perceived gratitude, values alignment,[2] and supportive leadership), "Efficiency of Practice" (for the current study: Electronic Health Record [EHR] helpfulness and EHR hassles) and Personal Resilience (for the current study: self-valuation,[3] sleep-related impairment,[4] and impact of work on personal relationships[5]).

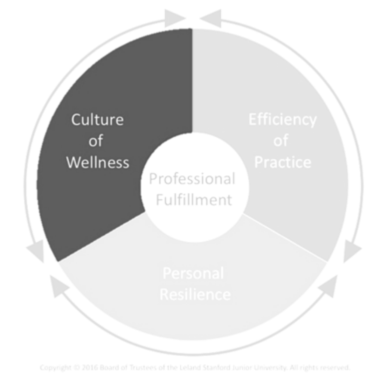

Culture of Wellness: Organizational values, behaviors and leadership that prioritize personal and professional growth, community, and compassion for self and others.[1,6–8]

A. Peer Support [9]
B. Control over Schedule [9]
C. Perceived Gratitude[9,10]
D. Personal/Organizational Values Alignment[2,9]
E. Supportive Leadership Behaviors[9,11]

<u>A. Peer Support:</u> How true are the following statements about <u>your peers at work</u>? [9]

**My peers at work...** [Score=]	**Not at all true** [0]	**Somewhat true** [1]	**Moderately true** [2]	**Very true** [3]	**Completely true** [4]
a. Listen empathetically when I talk about work-related stress	[ ]	[ ]	[ ]	[ ]	[ ]
b. Lift me up when I'm having a difficult day	[ ]	[ ]	[ ]	[ ]	[ ]
c. Help me find solutions to work problems	[ ]	[ ]	[ ]	[ ]	[ ]
d. Pitch in when I need help with my work	[ ]	[ ]	[ ]	[ ]	[ ]

<u>B. Control Over Schedule:</u> In your practice setting, how much control do you have over aspects of your work? [9]

**I have control over…** [Score=]	**No control** [0]	**A little control** [1]	**Moderate control** [2]	**A lot of control** [3]	**Complete control** [4]
a. The number of hours I work	[ ]	[ ]	[ ]	[ ]	[ ]
b. The schedule of hours I work	[ ]	[ ]	[ ]	[ ]	[ ]
c. Work interruptions (e.g., telephone calls, unscheduled patients)	[ ]	[ ]	[ ]	[ ]	[ ]

d. The volume of my patient load or panel size	[ ]	[ ]	[ ]	[ ]	[ ]
e. Last-minute schedule changes for unexpected personal needs (for family or self)	[ ]	[ ]	[ ]	[ ]	[ ]

C. Perceived Gratitude: Answer the following questions based on your experience during the past two weeks.[9,10]

**My colleagues and coworkers appreciate…** [Score=]	**Not at all** [0]	**A little bit** [1]	**Moderatel y** [2]	**Quite a bit** [3]	**Extremely** [4]
a. The work I do for my patients	[ ]	[ ]	[ ]	[ ]	[ ]
b. My contributions to our team	[ ]	[ ]	[ ]	[ ]	[ ]
c. Things I do for them	[ ]	[ ]	[ ]	[ ]	[ ]
d. Having me as a colleague or coworker	[ ]	[ ]	[ ]	[ ]	[ ]

D. Personal-Organizational Values Alignment: How true are the following statements about conditions in your practice setting (i.e., your principal practice site)? [2,9]

[Score=]	**Not at all true** [0]	**Somewhat true** [1]	**Moderately true** [2]	**Very true** [3]	**Completely true** [4]
a. My input is valued in important administrative decisions	[ ]	[ ]	[ ]	[ ]	[ ]
b. Our organizational goals and values fit well with my goals and values	[ ]	[ ]	[ ]	[ ]	[ ]
c. Administration values my clinical work	[ ]	[ ]	[ ]	[ ]	[ ]

F. **Supportive Leadership Behaviors:** assessed using a 9-item version of the 12-item Mayo Clinic Participatory Management Leadership Index that asks respondents to evaluate their immediate leader on measurable and actionable leadership behaviors that drive team engagement.[11] The measure has been linked with occupational well-being outcomes among physicians and those they lead.[2,11–13]

Efficiency of Practice: Workplace systems, processes, and practices that promote safety, quality, effectiveness, positive patient and colleague interactions, and work- life balance.[1,7,14]

G. EHR Helpfulness [9]
H. EHR Hassles [9]

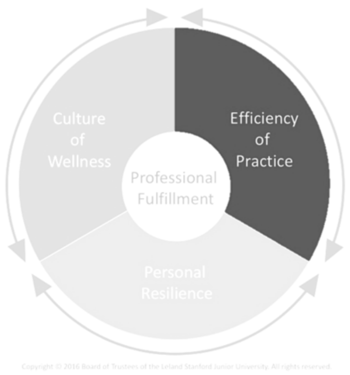

G. EHR Helpfulness: How often do you experience the following when using the Electronic Health Record (EHR)? [9]

[Score=]	**Never** [0]	**Rarely** [1]	**Sometimes** [2]	**Often** [3]	**Always** [4]
a. EHR tools help me communicate with patients efficiently	[ ]	[ ]	[ ]	[ ]	[ ]
b. I am able to quickly locate information I need	[ ]	[ ]	[ ]	[ ]	[ ]
c. EHR tools help me enter orders efficiently	[ ]	[ ]	[ ]	[ ]	[ ]
d. EHR tools help me coordinate care efficiently	[ ]	[ ]	[ ]	[ ]	[ ]

H. EHR Hassles: How often do you experience the following when using the Electronic Health Record (EHR)? [9]

[Score=]	**Never** [0]	**Rarely** [1]	**Sometimes** [2]	**Often** [3]	**Always** [4]
a. EHR work makes it hard for me to pay undivided attention to my patients during face-to-face visits	[ ]	[ ]	[ ]	[ ]	[ ]
b. I have to spend too much time completing EHR tasks other team members could do	[ ]	[ ]	[ ]	[ ]	[ ]
c. The amount of work I have to do in the EHR per patient is excessive	[ ]	[ ]	[ ]	[ ]	[ ]

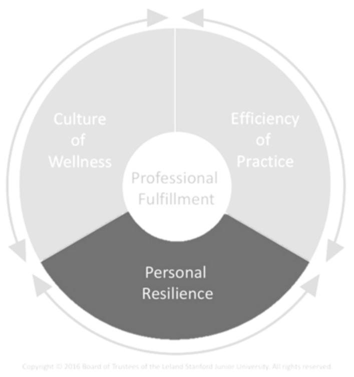

Personal Resilience: Individual skills, behaviors, and attitudes that contribute to physical, emotional, and professional well-being.[1,8,15–17]

I. Self-Valuation [3,9]
J. Sleep Related Impairment [4]
K. Adverse Impact of Work on Personal Relationships [5,9]

Self-Valuation: How often have you experienced the following? [3,9]

**During the past two weeks…** [Score=]	**Never** [4]	**Rarely** [3]	**Sometimes** [2]	**Often** [1]	**Always** [0]
a. When I made a mistake, I felt more self-condemnation than self-encouragement to learn from the experience	[ ]	[ ]	[ ]	[ ]	[ ]
b. I was less compassionate with myself than I was with others	[ ]	[ ]	[ ]	[ ]	[ ]

c. I put off taking care of my own health due to time pressure	[ ]	[ ]	[ ]	[ ]	[ ]
d. Taking care of my needs seemed incompatible with taking care of my patients' needs	[ ]	[ ]	[ ]	[ ]	[ ]

Scores ≥ 9 are favorable.[3] Previous research suggests self-valuation is lower in physicians than the general population and associated with several occupational well-being metrics in medicine.[3,13,18–21]

Sleep-Related Impairment: assessed using an 8-item short form version of the Patient-Reported Outcomes Measurement Information System Sleep-Related Impairment measure[22] developed by the National Institutes of Health (NIH PROMIS® Item Bank). [4,23–25] Respondents use a Likert scale ranging from "Not at all [0]" to "Very much [4] to describe their perception of alertness, sleepiness, tiredness, and associated functional limitations during waking hours in the past 7 days.[4] Sleep related impairment is associated with reduced occupational well-being in physicians.[5,20,26–28] A previous cross-sectional study found that higher sleep-related impairment scores (assessed using this 8-item version of the NIH PROMIS measure) were associated with increased odds of burnout, low professional fulfillment, and clinically significant medical errors among 11,395 US physicians.[19]

Negative Impact of Work on Personal Relationships: How has your job affected your personal relationships during the past year? [5]

**In the past year, my job has…** [Score=]	**Not at all true** [0]	**Somewhat true** [1]	**Moderately true** [2]	**Very true** [3]	**Completely true** [4]
a. Made it harder for me to nurture existing personal relationships	[ ]	[ ]	[ ]	[ ]	[ ]
b. Made it harder for me develop new meaningful personal relationships	[ ]	[ ]	[ ]	[ ]	[ ]
c. Contributed to conflict in my personal relationship(s)	[ ]	[ ]	[ ]	[ ]	[ ]
d. Contributed to me feeling more isolated or detached from the people who are most important to me	[ ]	[ ]	[ ]	[ ]	[ ]

II. Qualitative Data Collection Instrument

The development of the semi-structured qualitative interview guide was informed by a literature search. A core team of Stanford study investigators reviewed and revised the resulting guide: 1) a physician-scientist/academic psychiatrist leading the study, 2) an oncologist leading efforts to explore and address burnout across specialties, 3) a doctoral scientist and specialist in qualitative research among physicians, and 4) a Social Science Research Professional. Upon reaching consensus, two 60-minute pilot interviews were conducted neurologist to inform further refinement of the interview guide prior to the enrollment of study participants. The resultant semi-structured guide is included below.

Semi-Structured Interview Guide

**Category**	**Question**
Appreciative Inquiry	Why did you decide to become a neurologist?
Values	How is the reality of the profession different from what you'd expected it to be?<br>What patient population do you particularly like to work with and why?<br>What values did you learn growing up that contribute to how you define success?<br>What practices did you develop in residency, both detrimental and constructive, that shaped your current mindset about what it means to be a successful neurologist?<br>What is one thing you wish you could change about yourself?
Experience at Work	What does a typical workweek look like for you?<br>What do you feel are the greatest challenges you experience at work?<br>What parts of your work are most meaningful to you?<br>What do you feel would need to change in order for your professional life to be more fulfilling?<br>Can you tell me if and how the COVID pandemic has affected your work?
Strategies Used	What helps you integrate or balance your personal and professional responsibilities?<br>What specific strategies do you use to maintain your own wellness?<br>What do you do when your workload becomes excessive or overwhelming?<br>How much sleep are you getting per night on average?<br>Have you ever made changes in your work schedule? What prompted this change? What were the results of making that change?<br>What advice would you give to your younger self?
Burnout	How do you define burnout?<br>Have you ever experienced burnout yourself?<br>If so, what helped you move through it?<br>If you had a trainee or close colleague who you feared was burning out, what advice would you give them for recovering their well-being?

Culture at Work	How would you describe your work culture?<br>How do you feel the wellness needs and professional experience in your work environment are different for men and women?<br>What values do you feel are most important to the leadership at your institution?
Concluding Question	What question do you wish I'd asked?

References

1. Bohman B, Dyrbye L, Sinsky CA, Linzer M, Olson K, Babbott S, Murphy ML, deVries PP, Hamidi MS, Trockel M. Physician Well-Being: The Reciprocity of Practice Efficiency, Culture of Wellness, and Personal Resilience. *NEJM Catalyst*. Published online August 7, 2017. doi:https://catalyst.nejm.org/doi/full/10.1056/CAT.17.0429

2. Shanafelt TD, Wang H, Leonard M, Hawn M, McKenna Q, Majzun R, Minor L, Trockel M. Assessment of the Association of Leadership Behaviors of Supervising Physicians With Personal-Organizational Values Alignment Among Staff Physicians. *JAMA Netw Open*. 2021;4(2):e2035622. doi:10/gpb9cx

3. Trockel MT, Hamidi MS, Menon NK, Rowe SG, Dudley JC, Stewart MT, Geisler CZ, Bohman BD, Shanafelt TD. Self-valuation: Attending to the Most Important Instrument in the Practice of Medicine. *Mayo Clinic Proceedings*. 2019;94(10):2022-2031. doi:10.1016/j.mayocp.2019.04.040

4. Yu L, Buysse DJ, Germain A, Moul DE, Stover A, Dodds NE, Johnston KL, Pilkonis PA. Development of short forms from the PROMIS™ sleep disturbance and sleep-related impairment item banks. *Behavioral sleep medicine*. 2012;10(1):6-24. doi:10/fx9b2b

5. Trockel J, Bohman B, Wang H, Cooper W, Welle D, Shanafelt TD. Assessment of the Relationship Between an Adverse Impact of Work on Physicians' Personal Relationships and Unsolicited Patient Complaints. *Mayo Clinic Proceedings*. 2022;97(9):1680-1691. doi:10.1016/j.mayocp.2022.03.005

6. Shanafelt TD, Schein E, Minor LB, Trockel M, Schein P, Kirch D. Healing the Professional Culture of Medicine. *Mayo Clin Proc*. 2019;94(8):1556-1566. doi:10/gj87hb

7. Tawfik DS, Profit J, Webber S, Shanafelt TD. Organizational factors affecting physician well-being. *Curr Treat Options Pediatr*. 2019;5(1):11-25. doi:10/ggcdxc

8. Shanafelt T, Trockel M, Ripp J, Murphy ML, Sandborg C, Bohman B. Building a Program on Well-Being: Key Design Considerations to Meet the Unique Needs of Each Organization. *Academic Medicine*. 2019;94(2):156-161. doi:10.1097/ACM.0000000000002415

9. Trockel MT. Survey is property of Stanford University. Copyright 2018 Mickey Trockel, MD, PhD at Stanford University. All rights reserved.

10. Trockel MT, Menon NK, Makowski MS, Wen LY, Roberts R, Bohman BD, Shanafelt TD. IMPACT: Evaluation of a Controlled Organizational Intervention Using Influential Peers to Promote Professional Fulfillment. *Mayo Clinic Proceedings*. 2023;98(1):75-87. doi:10.1016/j.mayocp.2022.06.035

11. Shanafelt TD, Gorringe G, Menaker R, Storz KA, Reeves D, Buskirk SJ, Sloan JA, Swensen SJ. Impact of organizational leadership on physician burnout and satisfaction. *Mayo Clinic proceedings*. 2015;90(4):432-440. doi:10/f26279

12. Mete M, Goldman C, Shanafelt T, Marchalik D. Impact of leadership behaviour on physician well-being, burnout, professional fulfilment and intent to leave: a multicentre cross-sectional survey study. *BMJ Open*. 2022;12(6):e057554. doi:10.1136/bmjopen-2021-057554

13. Shanafelt TD, Makowski MS, Wang H, Bohman B, Leonard M, Harrington RA, Minor L, Trockel M. Association of Burnout, Professional Fulfillment, and Self-care Practices of Physician Leaders With Their Independently Rated Leadership Effectiveness. *JAMA Netw Open*. 2020;3(6):e207961-e207961. doi:10/gpb9bp

14. Shanafelt TD, Dyrbye LN, Sinsky C, Hasan O, Satele D, Sloan J, West CP. Relationship Between Clerical Burden and Characteristics of the Electronic Environment With Physician Burnout and Professional Satisfaction. *Mayo Clin Proc*. 2016;91(7):836-848. doi:10/f8vrz2

15. Brazeau CMLR, Trockel MT, Swensen SJ, Shanafelt TD. Designing and Building a Portfolio of Individual Support Resources for Physicians. *Academic Medicine*. Published online 2023. doi:10.1097/ACM.0000000000005276

16. Menon NK, Trockel MT, Hamidi MS, Shanafelt TD. Developing a Portfolio to Support Physicians' Efforts to Promote Well-being: One Piece of the Puzzle. *Mayo Clinic Proceedings*. 2019;94(11):2171-2177. doi:10/gpb9bt

17. West CP, Dyrbye LN, Sinsky C, Trockel M, Tutty M, Nedelec L, Carlasare LE, Shanafelt TD. Resilience and Burnout Among Physicians and the General US Working Population. *JAMA Network Open*. 2020;3(7):e209385-e209385. doi:10/gg44sw

18. Trockel M, Sinsky C, West CP, Dyrbye LN, Tutty M, Carlasare L, Wang H, Shanafelt T. Self-Valuation Challenges in the Culture and Practice of Medicine and Physician Well-being. *Mayo Clin Proc*. 2021;96(8):2123-2132. doi:10/gpb9c4

19. Trockel MT, Menon NK, Rowe SG, Stewart MT, Smith R, Lu M, Kim PK, Quinn MA, Lawrence E, Marchalik D, Farley H, Normand P, Felder M, Dudley JC, Shanafelt TD. Assessment of Physician Sleep and Wellness, Burnout, and Clinically Significant Medical Errors. *JAMA Network Open*. 2020;3(12):e2028111-e2028111. doi:10/gm3hbg

20. Marchalik D, Shaw N, Padmore J, Lu E, Rowe S, Trockel M. The impact of sleep-related impairment on burnout in urologists: Results from a national consortium study. *European Urology Supplements*. 2019;18. doi:10.1016/S1569-9056(19)30807-3

21. Shanafelt TD, Dyrbye LN, Sinsky C, Trockel M, Makowski MS, Tutty M, Wang H, Carlasare LE, West CP. Imposter Phenomenon in US Physicians Relative to the US Working Population. *Mayo Clinic Proceedings*. 2022;97(11):1981-1993. doi:10.1016/j.mayocp.2022.06.021

22. Cella D, Riley W, Stone A, Rothrock N, Reeve B, Yount S, Amtmann D, Bode R, Buysse D, Choi S. The Patient-Reported Outcomes Measurement Information System (PROMIS) developed and tested its first wave of adult self-reported health outcome item banks: 2005–2008. *Journal of clinical epidemiology*. 2010;63(11):1179-1194. doi:10/d8gnvj

23. HealthMeasures, U. S. Department of Health and Human Services. A brief guide to the Patient-Reported Outcomes Measurement Information System (PROMIS) Sleep-Related Impairment instruments.

24. Yu L, Buysse DJ, Germain A, Moul DE, Stover A, Dodds NE, Johnston KL, Pilkonis PA. Development of short forms from the PROMIS sleep disturbance and Sleep-Related Impairment item banks. *Behav Sleep Med*. 2011;10(1):6-24. doi:10/fx9b2b

25. Cella D, Choi SW, Condon DM, Schalet B, Hays RD, Rothrock NE, Yount S, Cook KF, Gershon RC, Amtmann D, DeWalt DA, Pilkonis PA, Stone AA, Weinfurt K, Reeve BB. PROMIS® Adult Health Profiles: Efficient Short-Form Measures of Seven Health Domains. *Value in Health: The Journal of the International Society for Pharmacoeconomics and Outcomes Research*. 2019;22(5):537-544. doi:10.1016/j.jval.2019.02.004

26. Trockel M, Bohman B, Lesure E, Hamidi MS, Welle D, Roberts L, Shanafelt T. A Brief Instrument to Assess Both Burnout and Professional Fulfillment in Physicians: Reliability and Validity, Including Correlation with Self-Reported Medical Errors, in a Sample of Resident and Practicing Physicians. *Acad Psychiatry*. Published online December 1, 2017. doi:10/gczbbn

27. Hamidi MS, Shanafelt TD, Hausel A, Bohman BD, Roberts R, Trockel MT. Associations Between Dietary Patterns and Sleep-Related Impairment in a Cohort of Community Physicians: A Cross-sectional Study. *American Journal of Lifestyle Medicine*. Published online September 10, 2019:1559827619871923. doi:10/gpb9bs

28. Welle D, Trockel MT, Hamidi MS, Hickson GB, Menon NK, Shanafelt TD, Cooper WO. Association of Occupational Distress and Sleep-Related Impairment in Physicians With Unsolicited Patient Complaints. *Mayo Clinic Proceedings*. 2020;95(4):719-726. doi:10/gpb9bw
